# Seven new species of *Pinelema* from Vietnam (Araneae, Telemidae)

**DOI:** 10.3897/zookeys.734.15061

**Published:** 2018-02-05

**Authors:** Huifeng Zhao, Pham Dinh-Sac, Yang Song, Thi-Duyen Do, Shuqiang Li

**Affiliations:** 1 Institute of Zoology, Chinese Academy of Sciences, Beijing 100101, China; 2 Southeast Asia Biodiversity Research Institute, Chinese Academy of Sciences, Yezin, Nay Pyi Taw 05282, Myanmar; 3 Graduate University of Science and Technology (GUST), Vietnam Academy of Science and Technology (VAST), Cau Giay, Hanoi, Vietnam; 4 School of Life and Environment Sciences, Gannan Normal University, Ganzhou, Jiangxi 341000, China; 5 University of Chinese Academy of Sciences, Beijing 100049, China

**Keywords:** haplogynae, karst, Southeast Asia, taxonomy

## Abstract

Seven new species of the spider genus *Pinelema* Wang & Li, 2012, from Vietnam are reported: *P. damtaoensis* Zhao & Li, **sp. n.** (♂♀), *P. nuocnutensis* Zhao & Li, **sp. n.** (♂♀), *P. laensis* Zhao & Li, **sp. n.** (♂♀), *P. pacchanensis* Zhao & Li, **sp. n.** (♂♀), *P. spirulata* Zhao & Li, **sp. n.** (♂♀), *P. xiezi* Zhao & Li, **sp. n.** (♂♀), and *P. zhenzhuang* Zhao & Li, **sp. n.** (♂♀). Prior to the current study, this genus contained eight species and was known only from southwestern China. The diagnosis of the genus is updated, accounting for characters found in the new species.

## Introduction

The spider family Telemidae Fage, 1913 contains nine genera and 69 species (World Spider Catalog 2017). *Pinelema* Wang & Li, 2012 is the second-most species rich genus of the family, comprising eight species from the Yunnan-Guizhou Plateau of China. Here, seven new species of *Pinelema* are described from Vietnam. The diagnosis and description of the genus are extended. Prior to this study, only two species of the spider family Telemidae were known from Vietnam: *Telema cucphongensis* Lin, Pham & Li, 2009 and *T. exiloculata* Lin, Pham & Li, 2009 (Lin et al. 2009).

## Materials and methods

**Figure 1.**
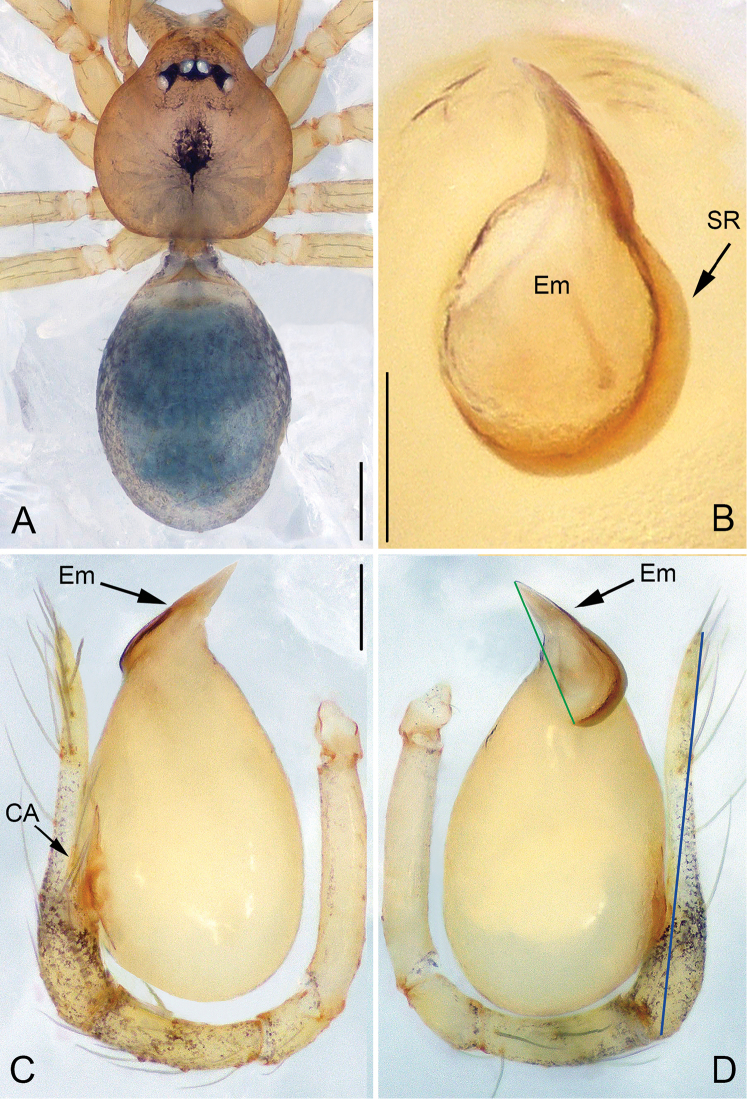
*Pinelema damtaoensis* sp. n., male holotype. **A** Habitus, dorsal view **B** Embolus, apical view **C** Palp, prolateral view **D** Palp, retrolateral view. Scale bars: 0.2 mm (**A**), 0.01 mm (**B**), 0.1 mm (**C–D**). Green line indicates the length of embolus; blue line indicates the length of cymbium.

All specimens were examined and measured using a LEICA M205 C stereomicroscope. The bodies, male palps, and receptacles were photographed using an Olympus C7070 digital camera. Images were combined using Helicon Focus version 6.7.1 image stacking software (http://www.heliconsoft.com). Endogynes were removed and treated in lactic acid before photographing. All measurements are given in millimeters. Leg measurements are shown as: total length (femur, patella, tibia, metatarsus, tarsus). The left palpi of males were photographed using an FEI Quanta 450 Environmental Scanning Electron Microscope. The following abbreviations are used in the text and figures:

**CA** cymbial apophysis;

**Em** embolus;

**Re** receptacle;

**REC** the ratio of embolus length (green line in Fig. 1D) and cymbium length (blue line in Fig. 1D);

**SR** spiral ridge of embolus.

All specimens treated here are deposited in the Institute of Zoology, Chinese Academy of Sciences (IZCAS), Beijing, China.

## Taxonomy

### Family Telemidae Fage, 1913

#### Genus Pinelema

Taxon classificationAnimaliaORDOFAMILIA

Wang & Li, 2012

##### Type species.

*Pinelema bailongensis* Wang & Li, 2012 from Guangxi, China.

##### Diagnosis.

*Pinelema* is similar to *Telema* Simon, 1882 and can be distinguished from *Telema* by the presence of a distinct cymbial apophysis that is lacking in *Telema* (Wang et al. 2012, figs 2C, 4A).

##### Comments.

*Pinelema* species are small (0.97–1.80). Carapace 0.48–0.75 long, yellow, with long thin legs relative to body length; tibia I 0.94–2.08 long. Six eyes are normally developed, vestigial, or in some species are completely absent. If eyes are present, they are encircled by black rings. Male palps are large relative to their body, with a distinct cymbial apophysis; embolus is long, medium or short in comparison to the cymbium; the REC varies from 0.28 to 0.90. The receptacle is unpaired as in other telemids and has spiral ducts inside.

##### Distribution.

China, Vietnam.

##### Natural history.

*Pinelema* species inhabit karst caves or leaf litter of tropical rainforests.

#### Pinelema damtaoensis

Taxon classificationAnimaliaAraneaeTelemidae

Zhao & Li sp. n.

http://zoobank.org/DBBB5FB5-90DB-43C6-9839-92C0031B75D7

Figs 1 , 2 , 3 , 22

##### Type material.

***Holotype*** ♂: Vietnam: Vinh Phuc Province: Dam Tao National Park: N21°27.62', E105°38.91', 999 m, leaf litter, 1.XI.2012, H.F. Zhao & Z.G. Chen leg. ***Paratypes*** 3♂ and 5♀, same data as holotype.

##### Etymology.

The specific name refers to the type locality; adjective.

##### Diagnosis.

**Figure 2.**
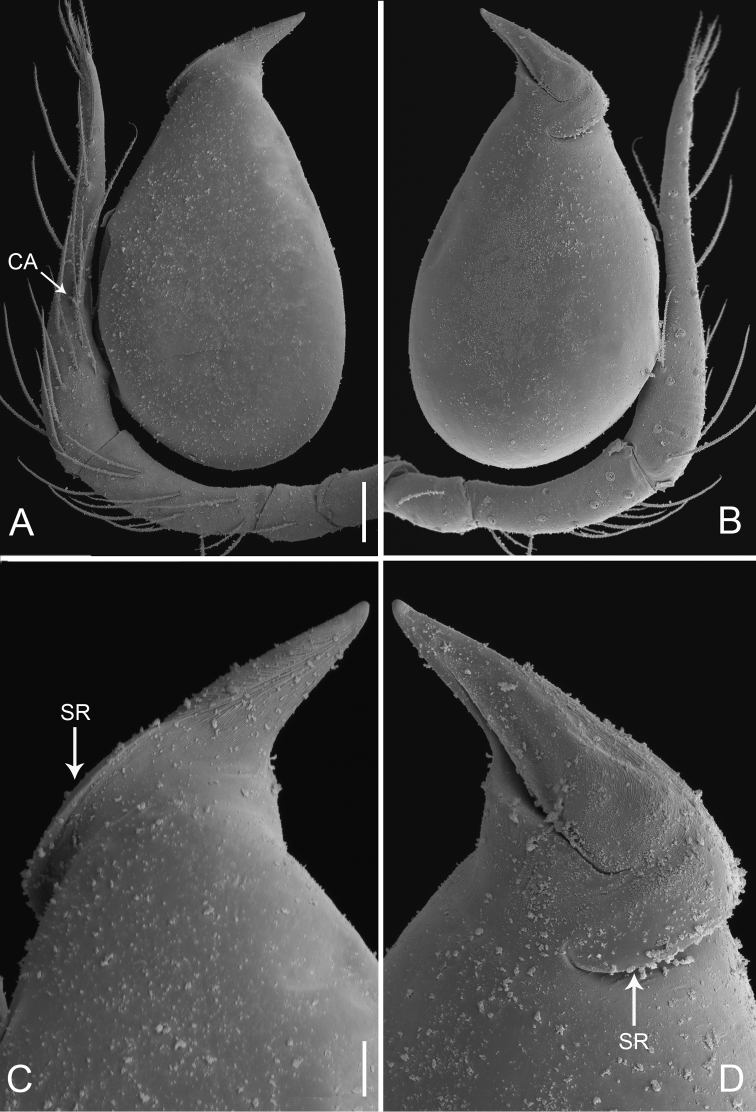
*Pinelema damtaoensis* sp. n., male. **A** Palp, prolateral view **B** Palp, retrolateral view **C** Embolus, prolateral view **D** Embolus, retrolateral view. Scale bars: 0.06 mm (**A–B**), 0.02 mm (**C–D**).

The new species is similar to *P. huobaensis* and *P. yaosaensis* by having a short, triangular embolus, but can be distinguished from them by a distinct brown spiral ridge (Figs 1B, 1D, 2C–D) and droplet-shaped bulb (Figs 1C–D, 2A–B) vs. an indistinct spiral ridge of the embolus and an egg-shaped bulb in *P. huobaensis* and *P. yaosaensis*, as well as by a beak-shaped embolus (Figs 1B, 1D, 2D) vs. an equilateral triangular embolus in related species.

##### Description.

***Male* (holotype).** Total length 1.28. Carapace 0.53 long, 0.48 wide. Abdomen 0.68 long, 0.53 wide. Carapace light brown with a black spot and radial stripes (Fig. 2A). Sternum dark brown. Six eyes encircled by black rings, well-developed, clypeus 0.08 long, ocular quadrangle 0.18 wide. Leg measurements: I 3.55 (1.01, 0.19, 1.05, 0.74, 0.56); II 2.97 (0.86, 0.19, 0.85, 0.59, 0.48); III 2.12 (0.64, 0.16, 0.56, 0.37, 0.39); IV 2.61 (0.81, 0.18, 0.71, 0.51, 0.40). Abdomen blue-green, with sparse long hairs.

Palp: femur 2.7 times longer than patella, tibia 2.5 times longer than patella, cymbial apophysis brown and spine-like (Figs 1C, 2A); REC 0.40; bulb droplet-shaped; embolus beak-shaped (Figs 1C–D, 2A–D), spiral ridge dark brown, continuing approximately 180° around embolus (Figs 1B–D, 2B–D), opening of embolus slit-like, extending from the base to the tip (Fig. 2D).

**Figure 3.**
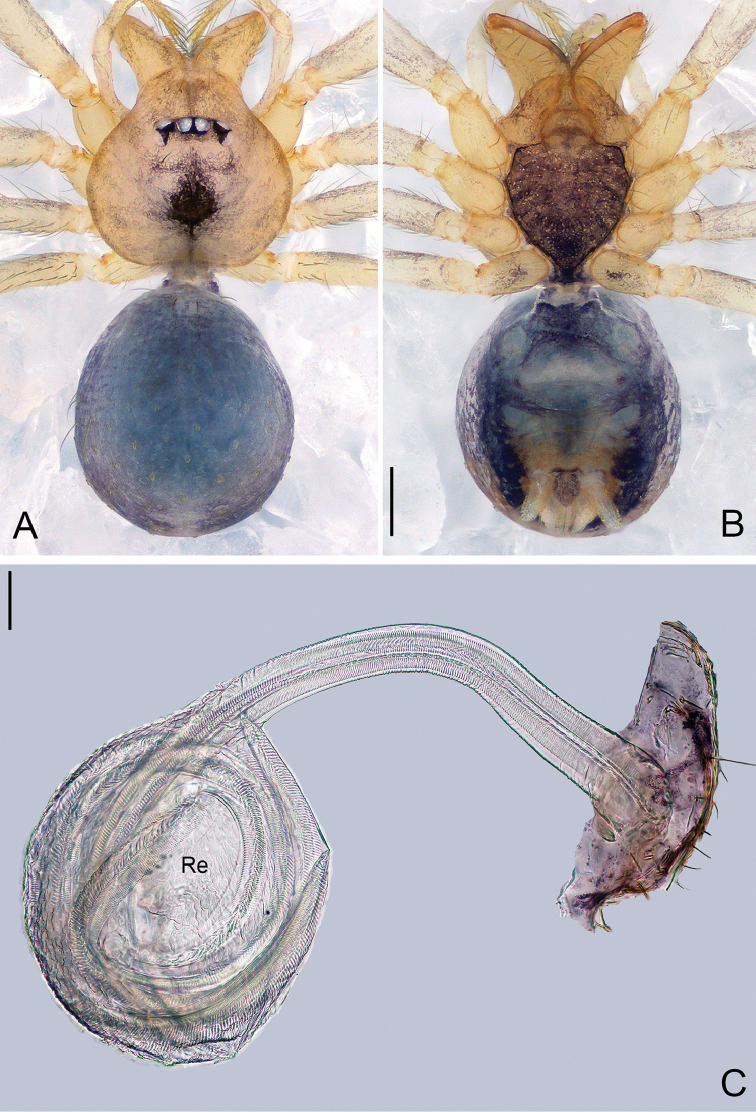
*Pinelema damtaoensis* sp. n., female paratype. **A** Habitus, dorsal view **B** Habitus, ventral view **C** Endogyne, lateral view. Scale bars: 0.2 mm (**A–B**), 0.05 mm (**C**).

***Female.*** Total length 1.39. Carapace 0.59 long, 0.55 wide. Abdomen 0.71 long, 0.59 wide. Coloration same as in male (Figs 3A–B). Six eyes, well-developed, clypeus 0.14 long, ocular quadrangle 0.21 wide. Leg measurements: I 3.77 (1.13, 0.19, 1.14, 0.75, 0.56); II 3.00 (0.92, 0.17, 0.90, 0.56, 0.45); III 2.28 (0.73, 0.16, 0.63, 0.40, 0.36); IV 2.92 (0.96, 0.16, 0.82, 0.57, 0.41). Receptacle globular, with long insemination duct, almost 2 times longer than receptacle diameter, and 5 times thinner than receptacle, receptacle diameter 0.14 wide (Fig. 3C).

##### Distribution.

Known only from the type locality (Fig. 22).

#### Pinelema nuocnutensis

Taxon classificationAnimaliaAraneaeTelemidae

Zhao & Li sp. n.

http://zoobank.org/86C258F1-260D-4009-83D9-4EC186BB7E08

Figs 4 , 5 , 6 , 22

##### Type material.

***Holotype*** ♂: Vietnam: Quang Binh Province: Phong Nha-Ke Bang National Park: Nuoc Nut Cave, N17°29.62', E106°17.65', 143 m, 25.V.2016, Z.G. Chen & Q.Y. Zhao leg. ***Paratypes*** 2♂ and 5♀, same data as holotype.

##### Etymology.

The specific name refers to the type locality; adjective.

##### Diagnosis.

**Figure 4.**
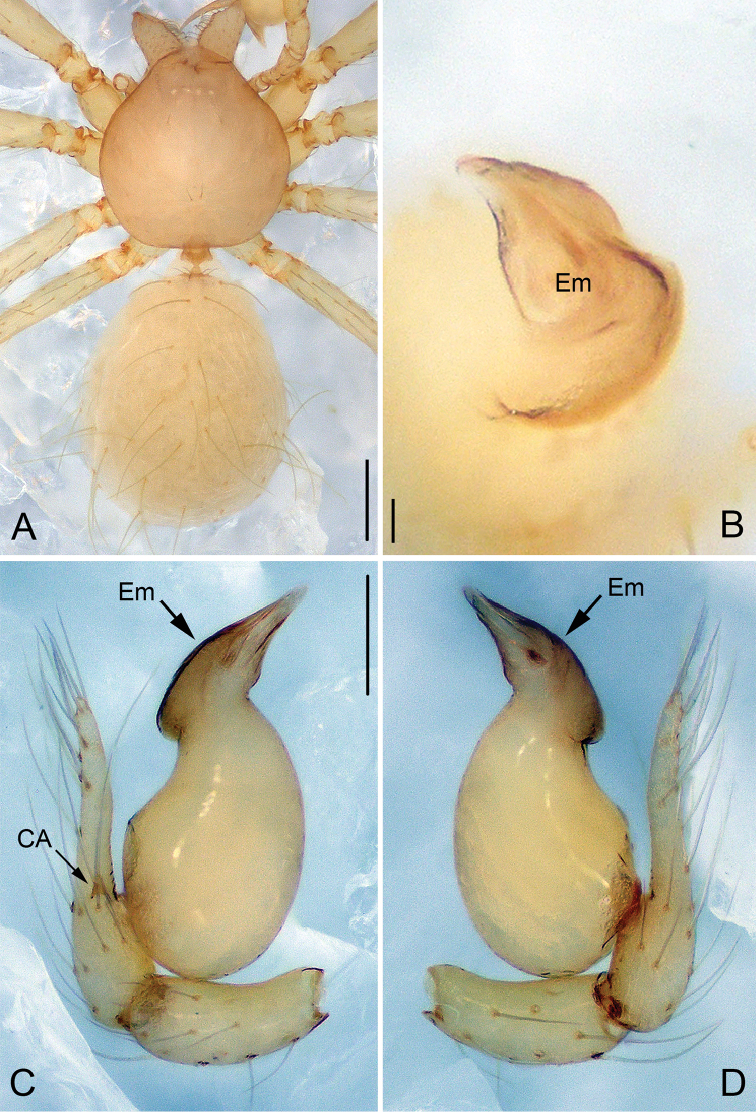
*Pinelema nuocnutensis* sp. n., male holotype. **A** Habitus, dorsal view **B** Embolus, apical view **C** Palp, prolateral view **D** Palp, retrolateral view. Scale bars: 0.2 mm (**A**), 0.02 mm (**B**), 0.1 mm (**C–D**).

**Figure 5.**
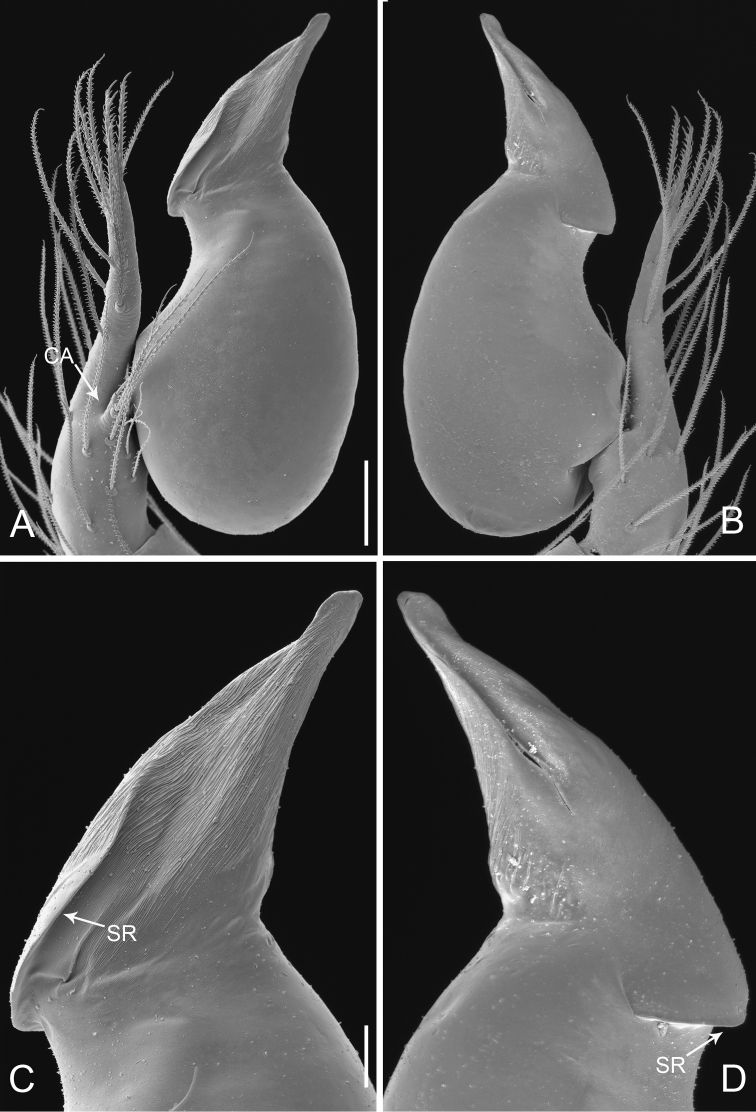
*Pinelema nuocnutensis* sp. n., male. **A** Palp, prolateral view **B** Palp, retrolateral view **C** Embolus, prolateral view **D** Embolus, retrolateral view. Scale bars: 0.06 mm (**A–B**), 0.02 mm (**C–D**).

**Figure 6.**
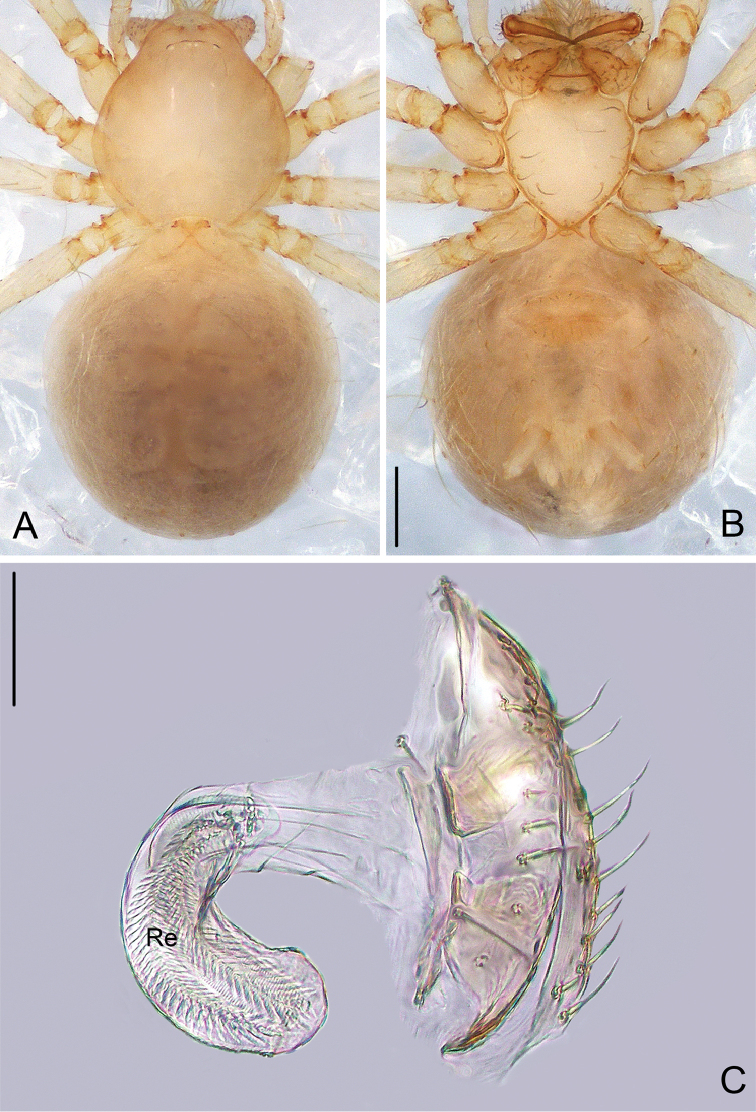
*Pinelema nuocnutensis* sp. n., female paratype. **A** Habitus, dorsal view **B** Habitus, ventral view **C** Endogyne, lateral view. Scale bars: 0.2 mm (**A–B**), 0.05 mm (**C**).

This new species is similar to *P. pacchanensis* sp. n., but can be distinguished by the vestigial eyes (Figs 4A, 6A) (eyes completely reduced in *P. pacchanensis* sp. n.), beak-shaped embolus (Figs 4B–D, 5A–D) (boomerang-shaped in *P. pacchanensis* sp. n.), and the U-shaped receptacle (Fig. 6C) (globular in *P. pacchanensis* sp. n.).

##### Description.

***Male* (holotype).** Total length 1.20. Carapace 0.53 long, 0.49 wide. Abdomen 0.63 long, 0.53 wide. Carapace, chelicerae, labium, and legs yellow, without any pattern (Fig. 4A). Four vestigial eyes. Leg measurements: I 4.42 (1.30, 0.20, 1.38, 0.90, 0.64); II 3.82 (1.13, 0.20, 1.18, 0.75, 0.56); III 2.73 (0.84, 0.18, 0.77, 0.51, 0.43); IV 3.24 (1.03, 0.18, 0.94, 0.63, 0.46). Abdomen light yellow, with a few long hairs.

Palp: femur 1.8 times longer than patella, tibia 1.5 times longer than patella, cymbial apophysis brown and spine-like (Figs 4C, 5A); REC 0.55; bulb kidney-shaped (Figs 4C–D, 5A–B); embolus beak-shaped, its outer margin forming a brown, spiral ridge (Fig. 4C–D, and arrows on Fig. 5C–D), opening two times shorter than embolus (Fig. 5B, D).

***Female.*** Total length 1.31. Carapace 0.54 long, 0.46 wide. Abdomen 0.81 long, 0.71 wide. Coloration and pattern as in male (Figs 6A–B). Eyes reduced to four vestigial spots. Leg measurements: I 4.18 (1.25, 0.19, 1.31, 0.80, 0.63); II 3.57 (1.06, 0.19, 1.08, 0.68, 0.56); III 2.68 (0.86, 0.16, 0.76, 0.48, 0.42); IV 3.14 (1.01, 0.17, 0.91, 0.59, 0.46). Endogyne J-shaped, with short and broad insemination duct and U-shaped receptacle, insemination duct as wide as receptacle. (Fig. 6C).

##### Distribution.

Known only from the type locality (Fig. 22).

#### Pinelema laensis

Taxon classificationAnimaliaAraneaeTelemidae

Zhao & Li sp. n.

http://zoobank.org/284193C9-50F3-4836-A3AC-B1C9790D49A9

Figs 7 , 8 , 9 , 22

##### Type material.

***Holotype*** ♂: Vietnam: Phu Tho Province: Xuan Son National Park: La Cave, N21°08.27', E104°56.35', 424 m, 27.X.2012, H.F. Zhao & Z.G. Chen leg. ***Paratypes*** 1♂ and 4♀, same data as holotype.

##### Etymology.

The specific name refers to the type locality; adjective.

##### Diagnosis.

**Figure 7.**
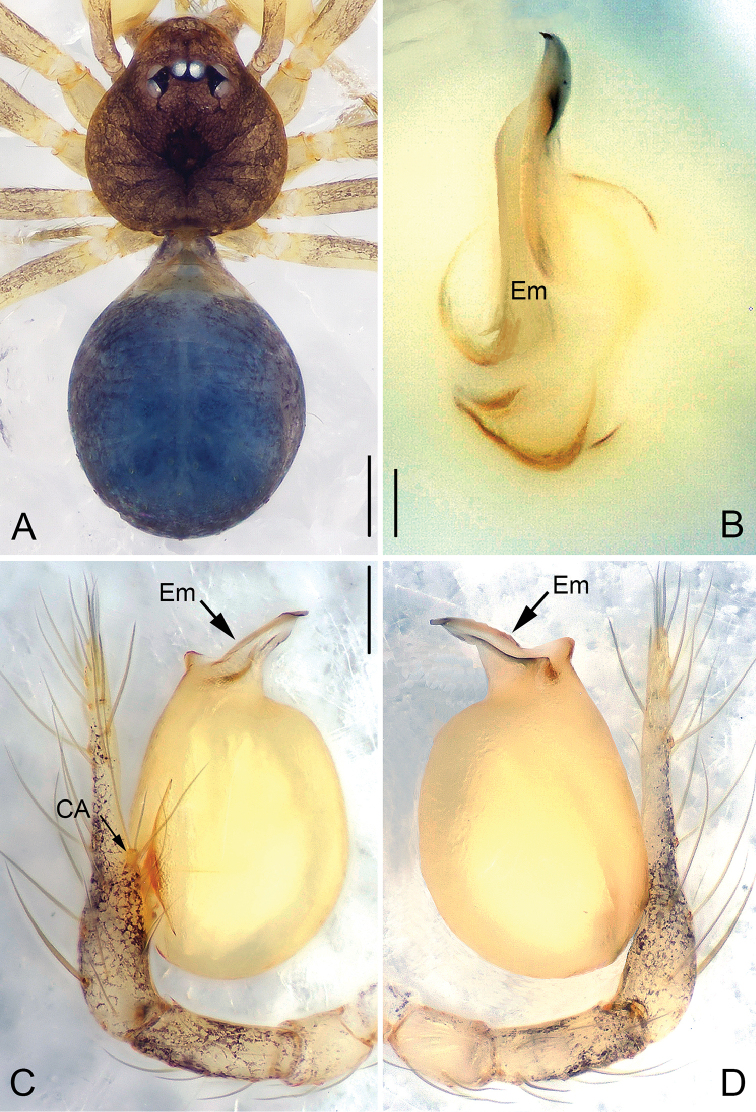
*Pinelema laensis* sp. n., male holotype. **A** Habitus, dorsal view **B** Embolus, apical view **C** Palp, prolateral view **D** Palp, retrolateral view. Scale bars: 0.2 mm (**A**), 0.02 mm (**B**), 0.1 mm (**C–D**).

**Figure 8.**
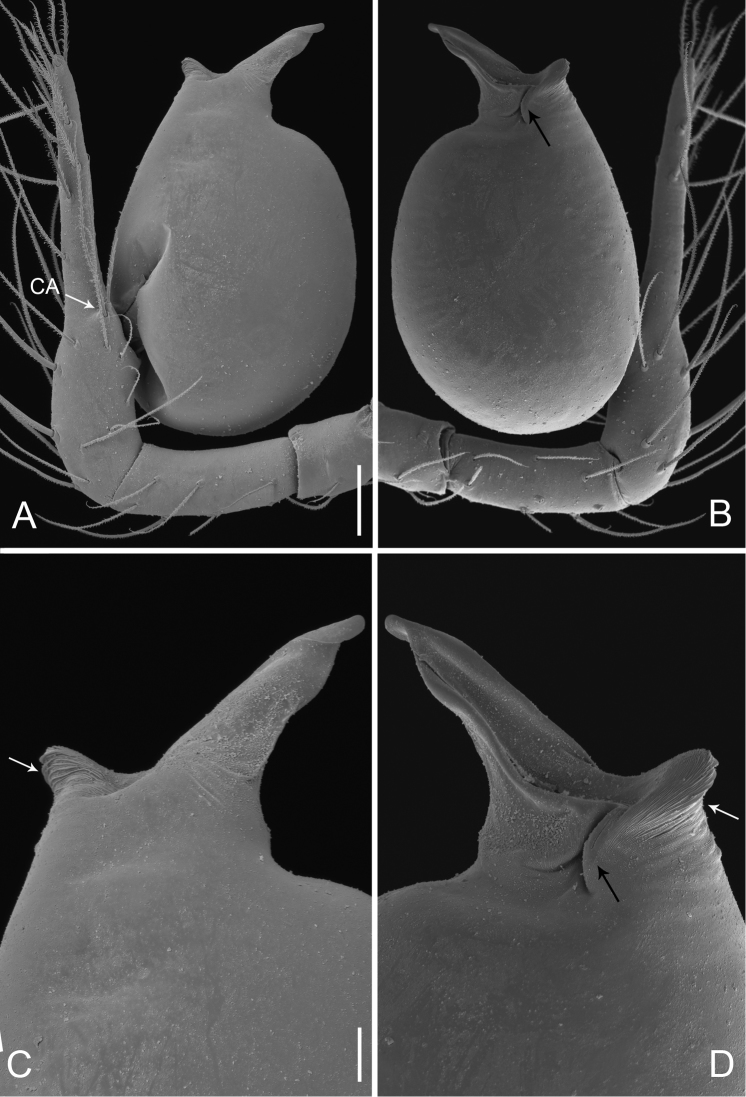
*Pinelema laensis* sp. n., male. **A** Palp, prolateral view **B** Palp, retrolateral view **C** Embolus, prolateral view **D** Embolus, retrolateral view. Scale bars: 0.06 mm (**A–B**), 0.02 mm (**C–D**). White arrows indicate tiny wrinkles and black ones indicate the groove of embolus.

**Figure 9.**
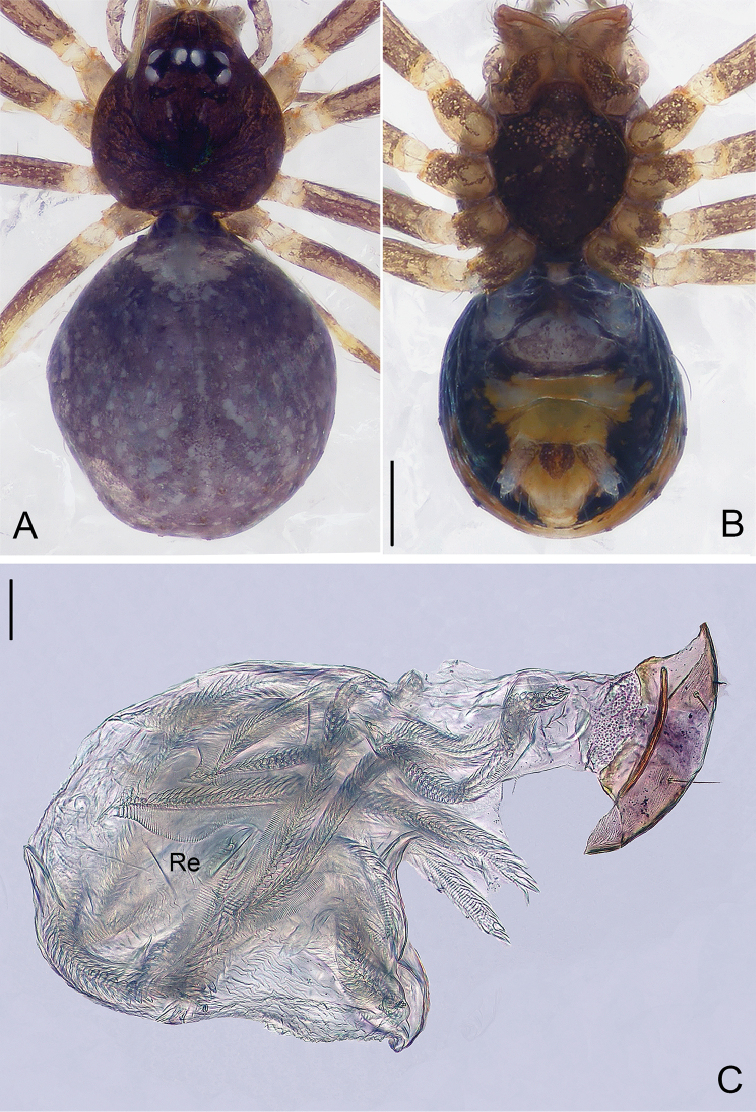
*Pinelema laensis* sp. n., female paratype. **A** Habitus, dorsal view **B** Habitus, ventral view **C** Endogyne, lateral view. Scale bars: 0.2 mm (**A–B**), 0.05 mm (**C**).

This new species is similar to *P. xiezi* sp. n. by having a trapezoidal embolus but can be distinguished by the marginally sclerotized, hollow embolus (Figs 7B, 8C–D). *Pinelema laensis* sp. n. is also similar to *P. huobaensis* and *P. yaosaensis* but can be distinguished from them by having distinct eyes and a trapezoidal embolus; *P. huobaensis* and *P. yaosaensis* have no eyes and their emboli are shaped like equilateral triangles. The new species can be distinguished from other congeners by the short embolus and axe-shaped endogyne (Fig. 9C).

##### Description.

***Male* (holotype).** Total length 1.20. Carapace 0.51 long, 0.47 wide. Abdomen 0.67 long, 0.55 wide. Carapace brown with a black spot. Six eyes encircled by black rings, clypeus 0.12 long, ocular quadrangle 0.19 wide. Chelicerae, labium and sternum dark brown. Legs yellow with brown dots and hairs (Fig. 7A), leg measurements: I 3.07 (0.87, 0.19, 0.94, 0.59, 0.48); II 2.66 (0.82, 0.15, 0.75, 0.48, 0.46); III 1.90 (0.56, 0.14, 0.52, 0.34, 0.34); IV 2.32 (0.71, 0.16, 0.64, 0.43, 0.38). Abdomen dark blue.

Palp: femur 2.5 times longer than patella, tibia approximately two times longer than patella, cymbial apophysis brown and thumb-like (Figs 7C, 8A); REC 0.56; bulb egg-shaped; embolus trapezoidal, sclerotized marginally, hollow inside (Figs 7C–D, 8C–D), slit of embolus obscure (Fig. 8D); embolus with vertical groove located retrolaterally (black arrows on Fig. 8B, D), with a pit prolaterally (Figs 7C, 8A, C) and wrinkles at the tip (white arrows on Fig. 8C–D).

***Female.*** Total length 1.19 (Figs 9A–B). Carapace 0.46 long, 0.45 wide. Abdomen 0.72 long, 0.69 wide. Coloration darker than male. Six eyes encircled by black rings, clypeus 0.09 long, ocular quadrangle 0.18 wide. Leg measurements: I 2.75 (0.82, 0.17, 0.81, 0.50, 0.45); II 2.41 (0.69, 0.17, 0.69, 0.43, 0.43); III 1.81 (0.54, 0.14, 0.50, 0.31, 0.32); IV 2.25 (0.71, 0.14, 0.64, 0.40, 0.36). Abdomen purple, with black and yellow pattern ventrally. Endogyne axe-shaped; insemination duct broad, its diameter 0.07; receptacle bag-like, 4 times wider than insemination duct (Fig. 9C).

##### Distribution.

Known only from the type locality (Fig. 22).

#### Pinelema pacchanensis

Taxon classificationAnimaliaAraneaeTelemidae

Zhao & Li sp. n.

http://zoobank.org/DA3352BD-B1B9-4215-A449-2D2A39BE4A3F

**Figure 10.**
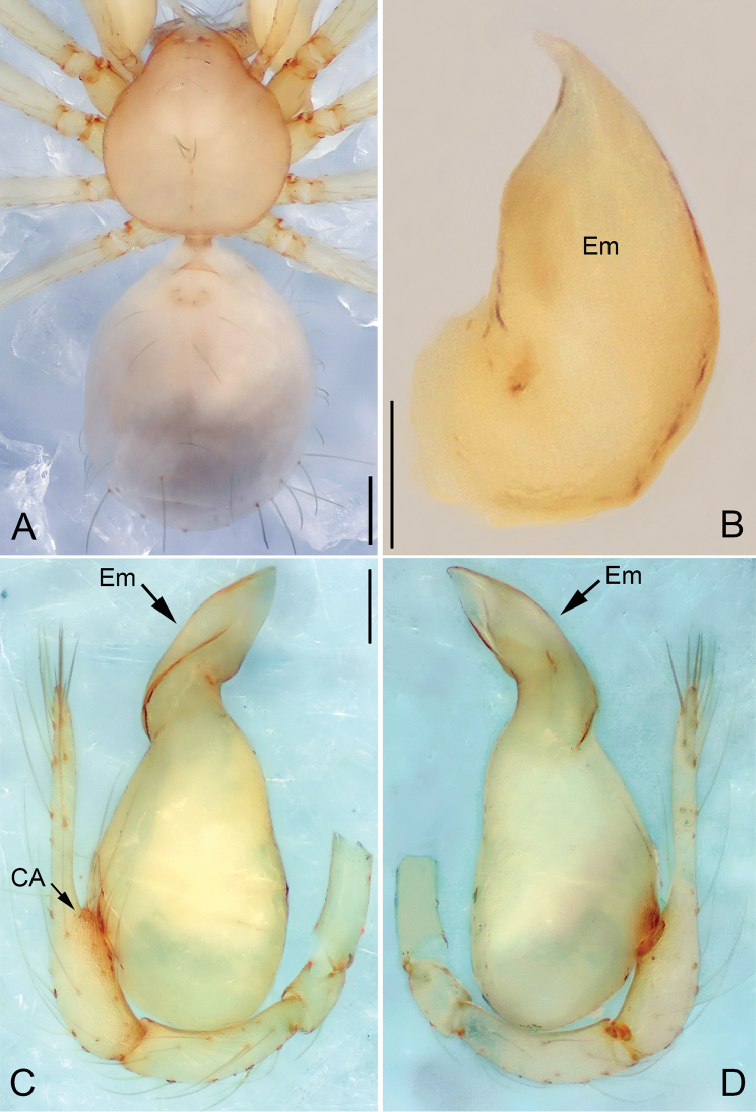
*Pinelema pacchanensis* sp. n., male holotype. **A** Habitus, dorsal view **B** Embolus, apical view **C** Palp, prolateral view **D** Palp, retrolateral view. Scale bars: 0.2 mm (**A**), 0.05 mm (**B**), 0.1 mm (**C–D**).

**Figure 11.**
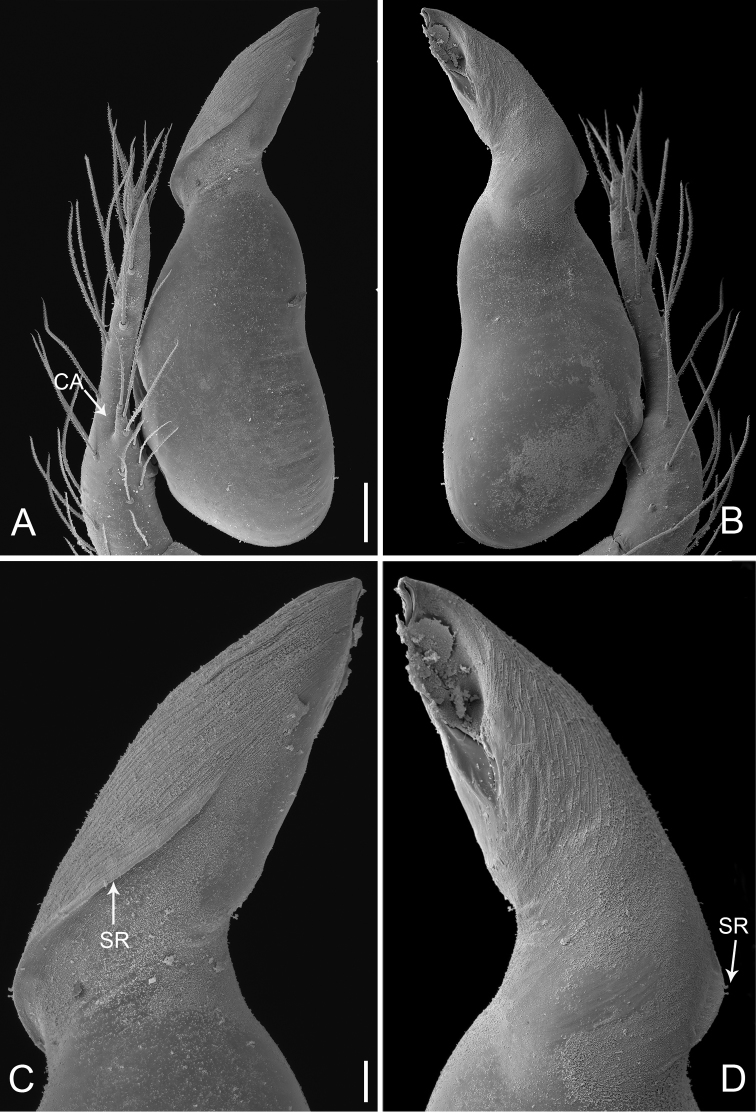
*Pinelema pacchanensis* sp. n., male. **A** Palp, prolateral view **B** Palp, retrolateral view **C** Embolus, prolateral view **D** Embolus, retrolateral view. Scale bars: 0.06 mm (**A–B**), 0.02 mm (**C–D**).

**Figure 12.**
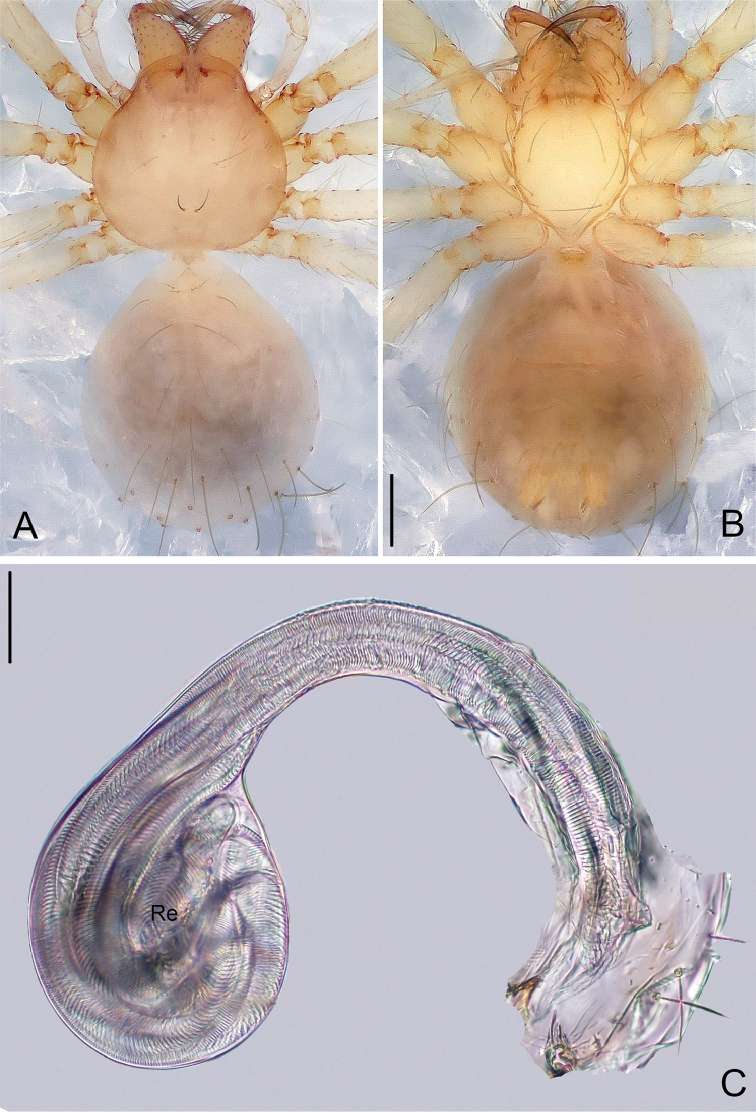
*Pinelema pacchanensis* sp. n., female paratype. **A** Habitus, dorsal view **B** Habitus, ventral view **C** Endogyne, lateral view. Scale bars: 0.5 mm (**A–B**), 0.05 mm (**C**).

Figs 10 , 11 , 12 , 22

##### Type material.

***Holotype*** ♂: Vietnam: Bac Kan Province: Cho Don District: Pac Chan Cave, N22°22.78', E105°36.79', 225 m, 18.X.2012, H.F. Zhao & Z.G. Chen leg. ***Paratypes*** 2♂ and 5♀, same data as holotype.

##### Etymology.

The specific name refers to the type locality; adjective.

##### Diagnosis.

This new species is similar to *P. nuocnutensis* sp. n. and *P. podiensis*, and can be distinguished from them by boomerang-shaped embolus (the embolus of *P. nuocnutensis* sp. n. is beak-shaped, embolus in *P. podiensis* is shaped like an isosceles triangle). The new species can be distinguished from other congeners by the medium length embolus (Figs 10C–D, 11A–B) (REC is 0.60) vs. REC of other *Pinelema* species 0.90 or less than 0.35.

##### Description.

***Male* (holotype).** Total length 1.41. Carapace 0.61 long, 0.55 wide. Abdomen 0.85 long, 0.71 wide. Carapace yellow with no markings. Eyes absent. Chelicerae, endites, labium, sternum, and legs the same color as carapace. Leg measurements: I 4.52 (1.34, 0.21, 1.41, 0.95, 0.61); II 4.13 (1.23, 0.21, 1.30, 0.83, 0.56); III 3.03 (0.95, 0.18, 0.85, 0.60, 0.45); IV 3.26 (1.18, 0.18, 1.08, 0.36, 0.46). Abdomen yellow with sparse long hairs.

Palp: femur approximately two times longer than patella, tibia nearly three times longer than patella, cymbium two times longer than tibia, cymbial apophysis brown and finger-shaped (Fig. 11A); REC 0.60; bulb kidney-shaped (Figs 10C–D, 11A–B); embolus boomerang-shaped (Figs 10D, 11D), spiral ridge brown (Figs 10B, 11C–D).

***Female.*** Total length 1.33 (Fig. 12A–B). Carapace 0.56 long, 0.55 wide. Abdomen 0.73 long, 0.67 wide. Coloration as in male. Leg measurements: I 4.26 (1.28, 0.22, 1.33, 0.86, 0.57); II 3.88 (1.18, 0.22, 1.19, 0.75, 0.54); III 2.89 (0.92, 0.16, 0.81, 0.54, 0.46); IV 3.56 (1.13, 0.17, 1.03, 0.75, 0.48). Endogyne comma-shaped, insemination duct long (2 times longer than diameter of receptacle) and its diameter twice as thin as receptacle. Receptacle globular (Fig. 12C).

##### Distribution.

Known only from the type locality (Fig. 22).

#### Pinelema spirulata

Taxon classificationAnimaliaAraneaeTelemidae

Zhao & Li sp. n.

http://zoobank.org/CAAA36BB-1471-4946-B8BF-657A483EA6BD

**Figure 13.**
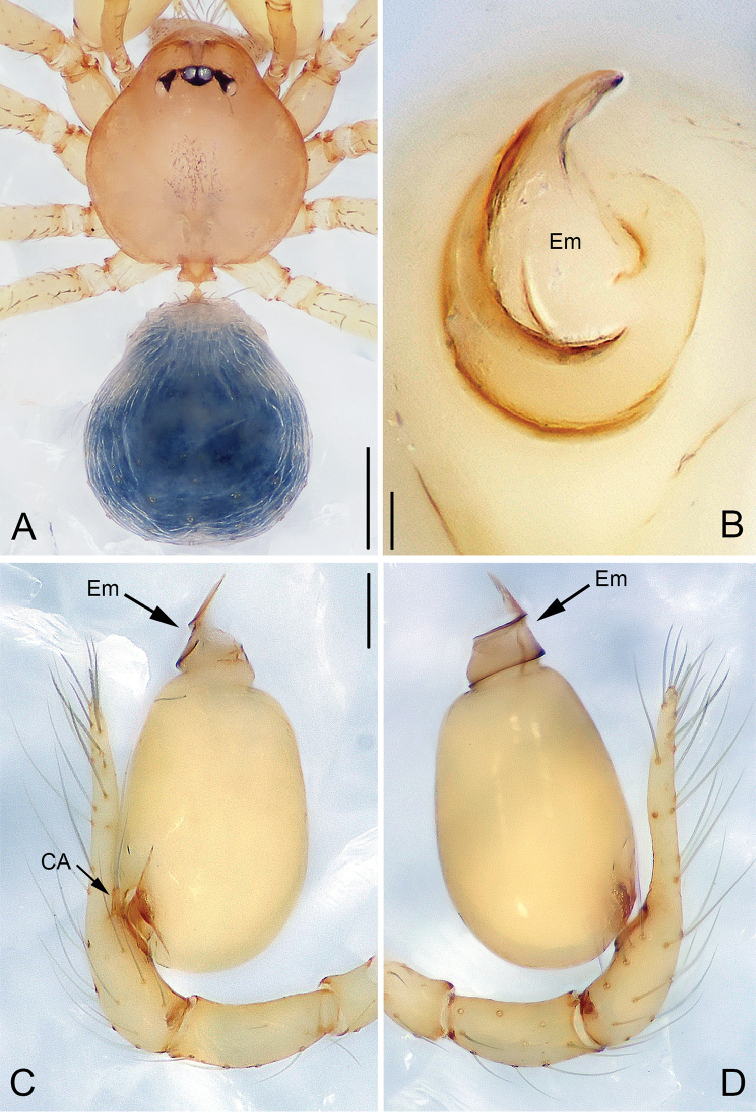
*Pinelema spirulata* sp. n., male holotype. **A** Habitus, dorsal view **B** Embolus, apical view **C** Palp, prolateral view **D** Palp, retrolateral view. Scale bars: 0.2 mm (**A**), 0.02 mm (**B**), 0.1 mm (**C–D**).

**Figure 14.**
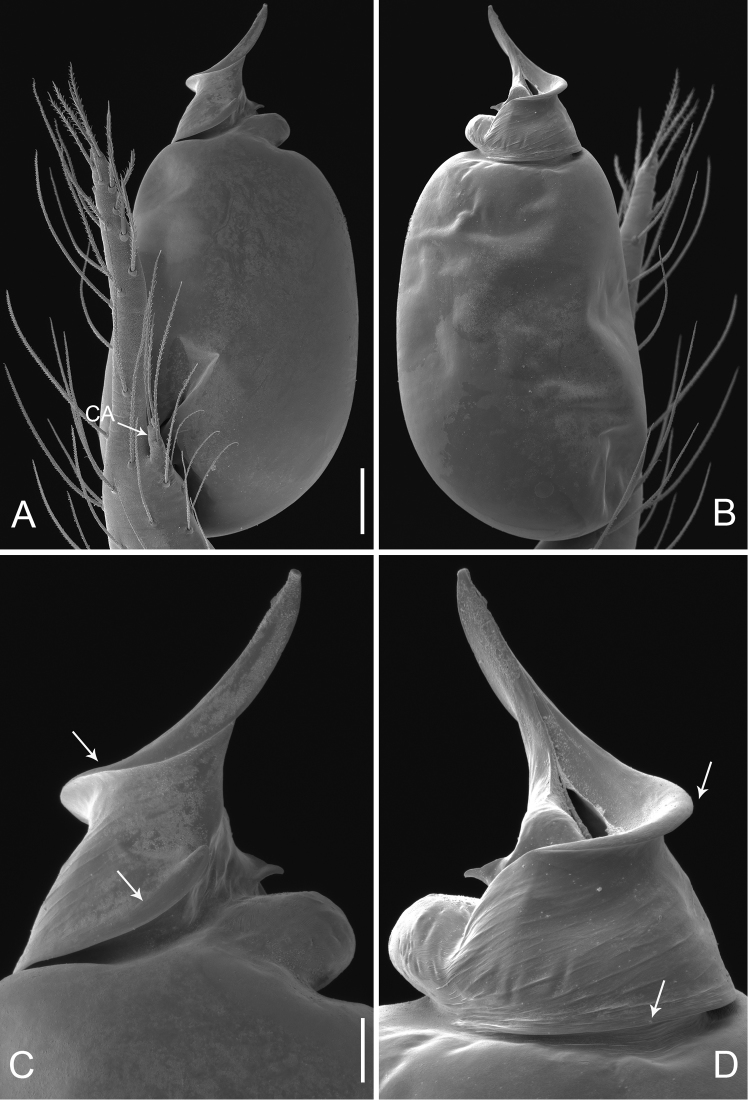
*Pinelema spirulata* sp. n., male. **A** Palp, prolateral view **B** Palp, retrolateral view **C** Embolus, prolateral view **D** Embolus, retrolateral view. Scale bars: 0.06 mm (**A–B**), 0.02 mm (**C–D**). Arrows indicate spiral ridge of embolus.

**Figure 15.**
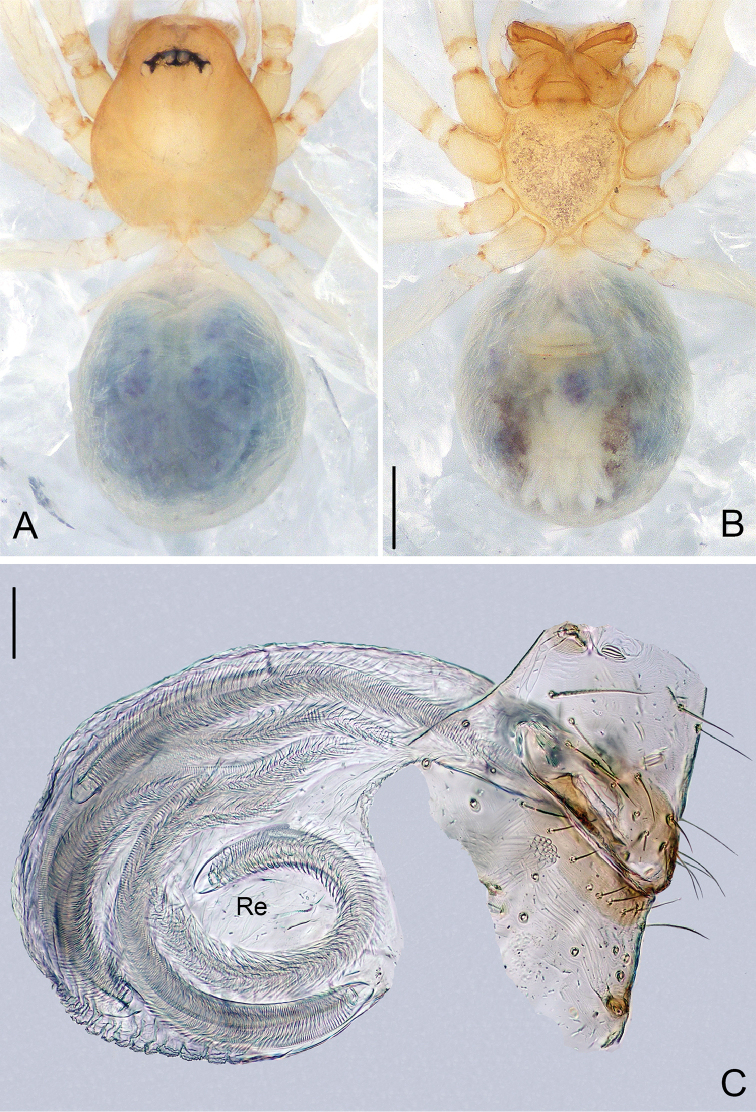
*Pinelema spirulata* sp. n., female paratype. **A** Habitus, dorsal view **B** Habitus, ventral view **C** Endogyne, lateral view. Scale bars: 0.2 mm (**A–B**), 0.1 mm (**C**).

Figs 13 , 14 , 15 , 22

##### Type material.

***Holotype*** ♂: Vietnam: Phu Tho Province: Xuan Son National Park: Lap Cave, N21°08.43', E104°56.57', 403 m, 2.X.2012, H.F. Zhao & Z.G. Chen leg. ***Paratypes*** 3♂ and 5♀, same data as holotype.

##### Etymology.

The specific name is derived from the Latin word “*spirulatus*”, meaning “screw-shaped”, and refers to the spiral embolus; adjective.

##### Diagnosis.

This new species can be distinguished from other congeners by the screw-shaped embolus (Figs 13B–D, 14A–D), emboli of other *Pinelema* species are either tube-shaped, triangular, or trapezoidal.

##### Description.

***Male* (holotype).** Total length 0.97. Carapace 0.51 long, 0.50 wide. Abdomen 0.50 long, 0.57 wide. Carapace yellow. Six eyes encircled by black rings, clypeus 0.08 long, ocular quadrangle 0.15 wide. Chelicerae, sternum, labium, and legs yellow. Leg measurements: I 3.71 (1.06, 0.19, 1.14, 0.76, 0.56); II 3.06 (0.90, 0.17, 0.91, 0.60, 0.48); III 2.25 (0.67, 0.16, 0.61, 0.42, 0.39); IV 2.58 (0.86, 0.16, 0.80, 0.39, 0.37). Abdomen dark blue with dense white hairs.

Palp: femur 2.5 times longer than patella, tibia approx. two times longer than patella, cymbium nearly two times longer than tibia, cymbial apophysis brown and spine like (Figs 13C, 14A); REC 0.28; bulb egg-shaped (Figs 13C–D, 14A–B); embolus spiral with brown ridge and tiny circular wrinkles (Figs 13B, 14C–D); the opening of embolus distinct (Fig. 14B, D).

***Female.*** Total length 1.30 (Fig. 15A–B). Carapace 0.52 long, 0.46 wide. Abdomen 0.74 long, 0.57 wide. Coloration as in male. Six eyes, clypeus 0.09 long, ocular quadrangle 0.17 wide. Leg measurements: I 3.50 (1.05, 0.14, 1.14, 0.65, 0.52); II 2.94 (0.87, 0.18, 0.90, 0.52, 0.47); III 2.23 (0.69, 0.16, 0.63, 0.39, 0.36); IV 2.77 (0.90, 0.16, 0.82, 0.49, 0.40). Endogyne as in Fig. 15C; insemination duct short, its diameter approx. 1/5 of receptacle diameter; receptacle with multiple membranous tubes inside.

##### Distribution.

Known only from the type locality (Fig. 22).

#### Pinelema xiezi

Taxon classificationAnimaliaAraneaeTelemidae

Zhao & Li sp. n.

http://zoobank.org/A6F02EFC-EFE5-47BF-B19F-07A517800007

**Figure 16.**
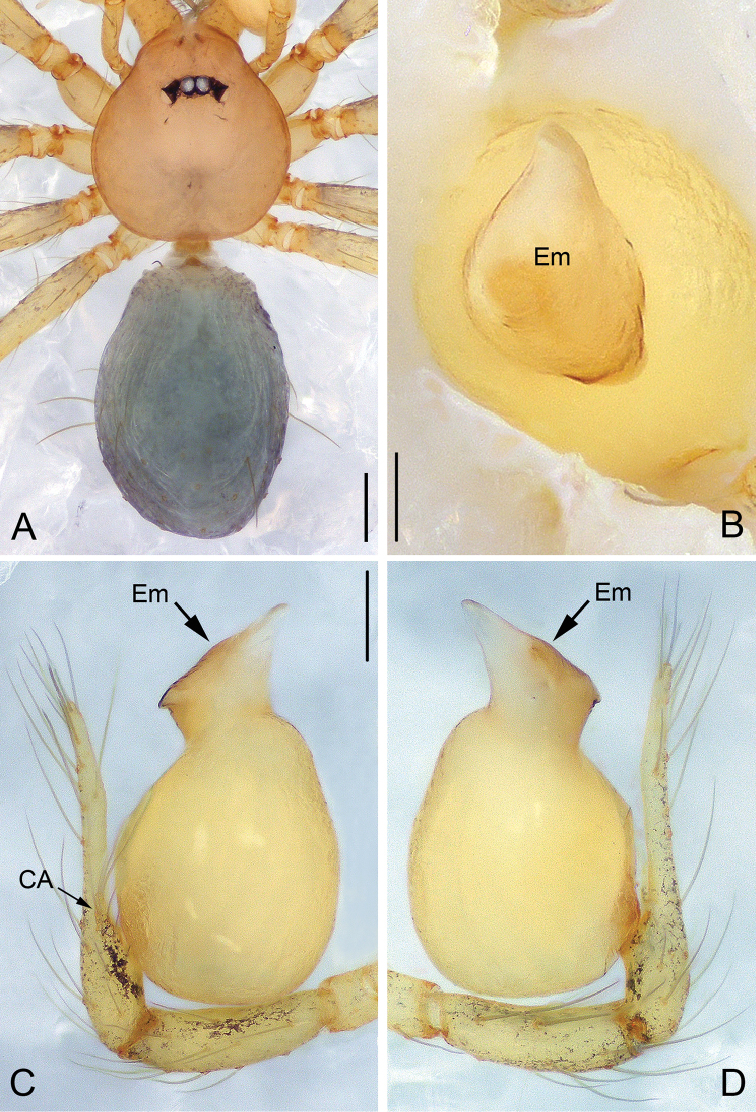
*Pinelema xiezi* sp. n., male holotype. **A** Habitus, dorsal view **B** Embolus, apical view **C** Palp, prolateral view **D** Palp, retrolateral view. Scale bars: 0.2 mm (**A**), 0.02 mm (**B**), 0.1 mm (**C–D**).

**Figure 17.**
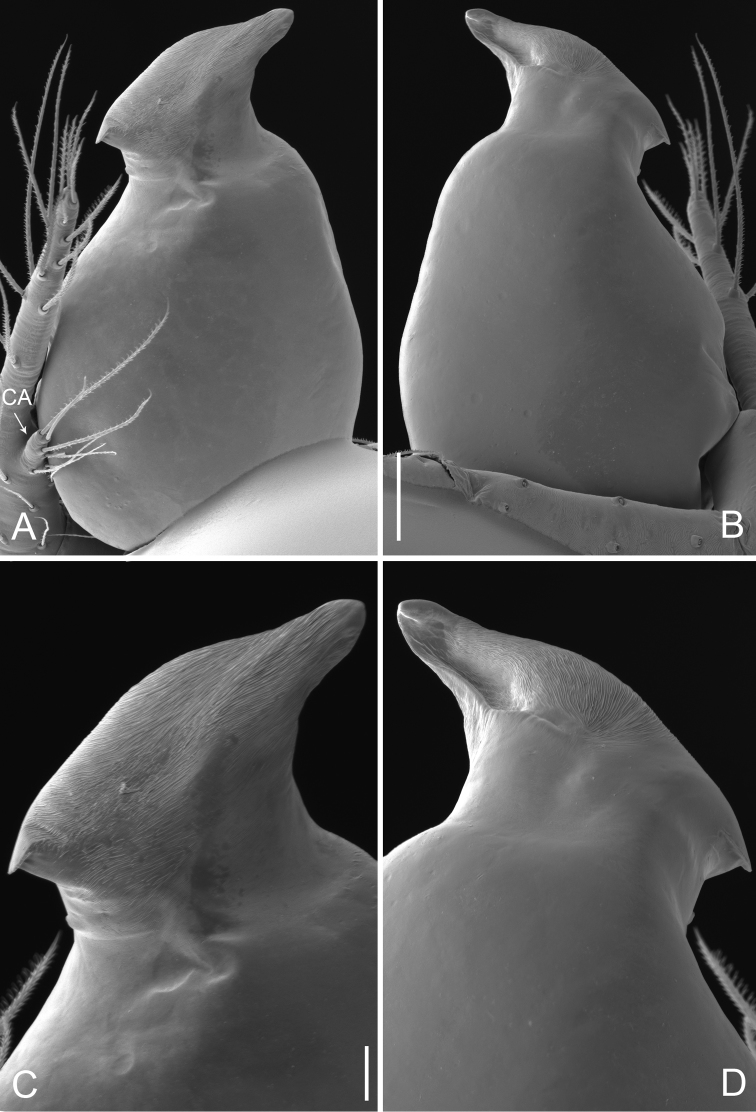
*Pinelema xiezi* sp. n., male. **A** Palp, prolateral view **B** Palp, retrolateral view **C** Embolus, prolateral view **D** Embolus, retrolateral view. Scale bars: 0.06 mm (**A–B**), 0.02 mm (**C–D**).

**Figure 18.**
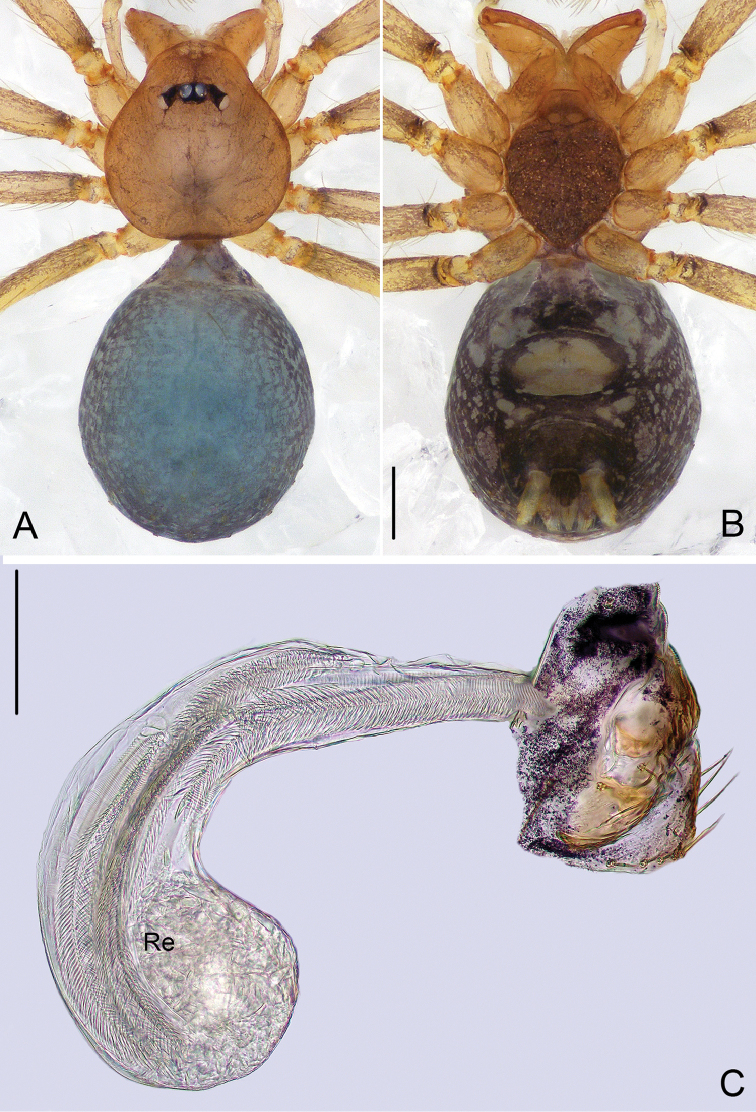
*Pinelema xiezi* sp. n., female paratype. **A** Habitus, dorsal view **B** Habitus, ventral view **C** Endogyne, lateral view. Scale bars: 0.2 mm (**A–B**), 0.05 mm (**C**).

Figs 16 , 17 , 18 , 22

##### Type material.

***Holotype*** ♂: Vietnam: Quang Binh Province: Phong Nha-Ke Bang National Park: Tien Son Cave, N17°34.80', E106°16.92', 102 m, 17.V.2016, Z.G. Chen & Q.Y. Zhao leg. ***Paratypes*** 3♂ and 5♀, same data as holotype.

##### Etymology.

This specific name is derived from the Chinese Pinyin ‘xié zǐ’, meaning ‘shoe’, in reference to the shoe-shaped embolus; noun.

##### Diagnosis.

This new species is similar to *P. huobaensis* and *P. yaosaensis* by a having short embolus. It can be distinguished from related species by the shoe-shaped embolus (Figs 16B, 17C–D) (vs. triangular embolus in related species). It is also similar to *P. laensis* sp. n. but can be distinguished by the unsclerotized margin of the embolus (the embolus of *P. laensis* sp. n. is sclerotized marginally and hollow). This new species can be distinguished from other congeners by the short embolus.

##### Description.

***Male* (holotype).** Total length 1.52. Carapace 0.63 long, 0.54 wide. Abdomen 0.75 long, 0.53 wide. Carapace, labium, sternum, and legs yellow. Six eyes encircled by black rings, clypeus 0.15 long, ocular quadrangle 0.19 wide. Leg measurements: I 4.63 (1.39, 0.22, 1.50, 0.92, 0.60); II 3.70 (0.95, 0.21, 1.25, 0.75, 0.54); III 2.86 (0.87, 0.21, 0.85, 0.53, 0.40); IV 3.45 (1.13, 0.19, 1.01, 0.67, 0.45). Abdomen long, elliptic, light blue with sparse long hairs.

Palp: femur 2.5 times longer than patella, tibia 2.2 times longer than patella, cymbial apophysis light yellow and finger-shaped (Figs 16C, 17A); REC 0.48; bulb yellow and egg-shaped; embolus shoe-shaped (Figs 16C–D, 17C–D), with a distinct groove at the tip (Fig. 17B, D) and tiny wrinkles (Fig. 17A–D).

***Female.*** Total length 1.38 (Fig. 18A–B). Carapace 0.57 long, 0.52 wide. Abdomen 0.79 long, 0.66 wide. Coloration same as in male. Six eyes, clypeus 0.12 long, ocular quadrangle 0.18 wide. Leg measurements: I 4.27 (1.33, 0.19, 1.36, 0.81, 0.58); II 3.60 (1.11, 0.21, 1.13, 0.65, 0.50); III 2.53 (0.79, 0.17, 0.72, 0.46, 0.39); IV 3.22 (1.06, 0.18, 0.92, 0.63, 0.43). Insemination duct thinner than receptacle, receptacle hockey stick-shaped (Fig. 18C).

##### Distribution.

Known only from the type locality (Fig. 22).

#### Pinelema zhenzhuang

Taxon classificationAnimaliaAraneaeTelemidae

Zhao & Li sp. n.

http://zoobank.org/BC2C4389-A40D-43C4-933D-8E035EC791B0

**Figure 19.**
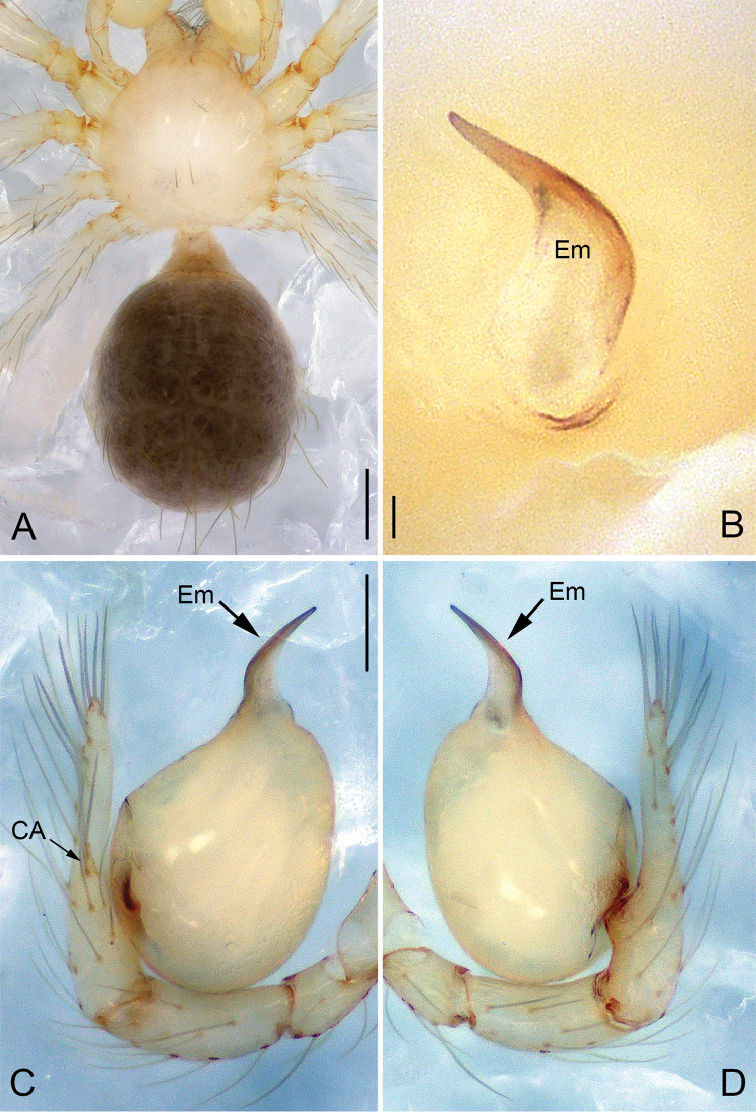
*Pinelema zhenzhuang* sp. n., male holotype. **A** Habitus, dorsal view **B** Embolus, apical view **C** Palp, prolateral view **D** Palp, retrolateral view. Scale bars: 0.2 mm (**A**), 0.02 mm (**B**), 0.1 mm (**C–D**).

**Figure 20.**
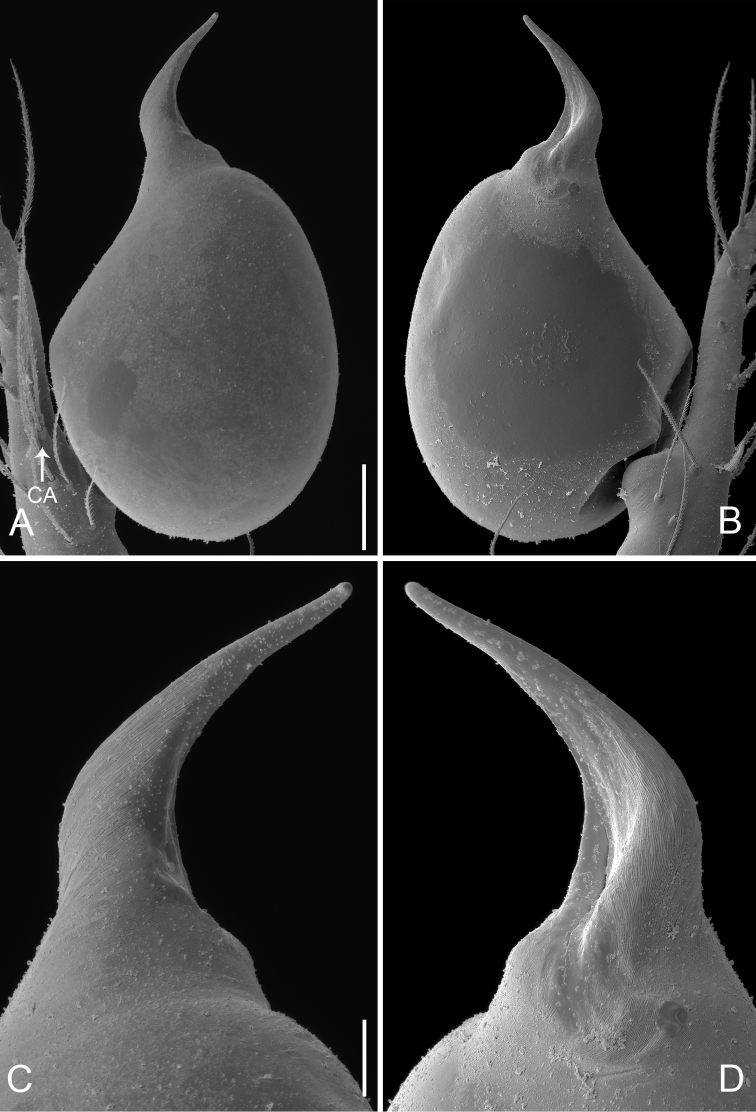
*Pinelema zhenzhuang* sp. n., male. **A** Palp, prolateral view **B** Palp, retrolateral view **C** Embolus, prolateral view **D** Embolus, retrolateral view. Scale bars: 0.06 mm (**A–B**), 0.02 mm (**C–D**).

**Figure 21.**
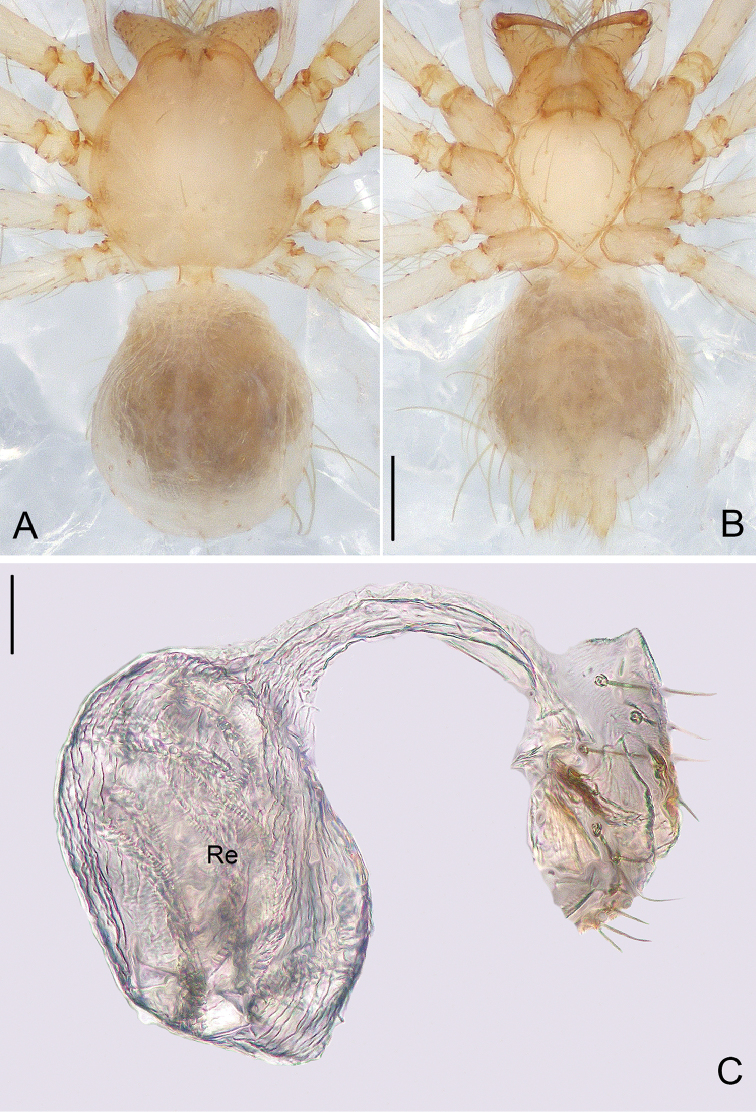
*Pinelema zhenzhuang* sp. n., female paratype. **A** Habitus, dorsal view **B** Habitus, ventral view **C** Endogyne, lateral view. Scale bars: 0.2 mm (**A–B**), 0.05 mm (**C**).

**Figure 22.**
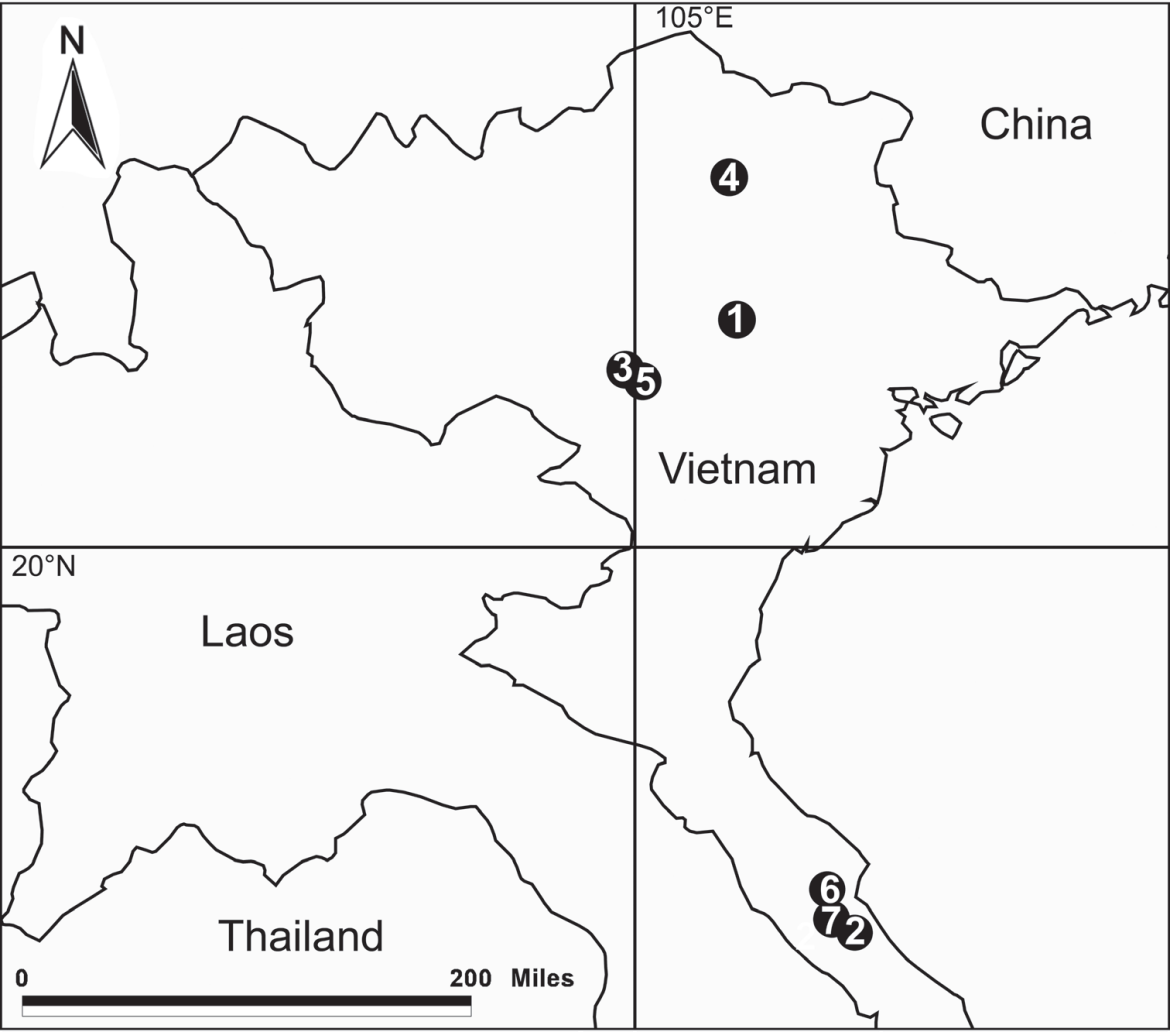
Distribution records of seven new *Pinelema* species in Vietnam: **1** *P. damtaoensis* sp. n. **2** *P. nuocnutensis* sp. n. **3** *P. laensis* sp. n. **4** *P. pacchanensis* sp. n. **5** *P. spirulata* sp. n. **6** *P. xiezi* sp. n. **7** *P. zhenzhuang* sp. n.

Figs 19 , 20 , 21 , 22

##### Type material.

***Holotype*** ♂: Vietnam: Quang Binh Province: Phong Nha-Ke Bang National Park: Tien Duong Cave, N17°31.17', E106°13.38', 133 m, 18.V.2016, Z.G. Chen & Q.Y. Zhao leg. ***Paratypes*** 3♂ and 4♀, same data as holotype.

##### Etymology.

This specific name is derived from the Chinese Pinyin ‘zhēn zhuàng’, meaning ‘needle-shaped’, in reference to the shape of the embolus; adjective.

##### Diagnosis.

This new species can be easily distinguished from other congeners by the short needle-shaped embolus (Figs 19B, 20C–D). The embolus of other *Pinelema* species is either long and tube-like, short and triangular or short and trapezoidal.

##### Description.

***Male* (holotype).** Total length 1.28. Carapace 0.49 long, 0.50 wide. Abdomen 0.69 long, 0.57 wide. Carapace yellow. Eyes absent. Chelicerae, sternum, and legs yellow. Leg measurements: I 4.80 (1.39, 0.22, 1.53, 0.99, 0.67); II 4.04 (1.20, 0.21, 1.28, 0.79, 0.56); III 2.93 (0.89, 0.18, 0.86, 0.54, 0.46); IV 3.44 (1.09, 0.19, 0.99, 0.71, 0.46). Abdomen brown with sparse long hairs.

Palp: femur 2.2 times longer than patella, tibia 1.8 times longer than patella, cymbial apophysis brown and spine-like (Figs 19C, 20A); REC 0.42; bulb acorn-shaped (Figs 19C–D, 20A–B); embolus bent and needle-shaped (Figs 19B–D, 20C–D), slit extending along entire embolus (Fig. 20B, D).

***Female.*** Total length 1.19 (Fig. 21A–B). Carapace 0.54 long, 0.51 wide. Abdomen 0.60 long, 0.53 wide. Coloration same as in male. Leg measurements: I 4.96 (1.50, 0.24, 1.56, 0.99, 0.67); II 4.25 (1.31, 0.22, 1.31, 0.83, 0.58); III 3.11 (0.99, 0.21, 0.90, 0.59, 0.42); IV 3.61 (1.20, 0.18, 1.05, 0.68, 0.50). Endogyne as in Fig. 21C; insemination duct narrow and its diameter shorter than length of receptacle; receptacle oval.

##### Distribution.

Known only from the type locality (Fig. 22).

## Supplementary Material

XML Treatment for Pinelema

XML Treatment for Pinelema damtaoensis

XML Treatment for Pinelema nuocnutensis

XML Treatment for Pinelema laensis

XML Treatment for Pinelema pacchanensis

XML Treatment for Pinelema spirulata

XML Treatment for Pinelema xiezi

XML Treatment for Pinelema zhenzhuang
